# Usage and comparison of artificial intelligence algorithms for determination of growth and development by cervical vertebrae stages in orthodontics

**DOI:** 10.1186/s40510-019-0295-8

**Published:** 2019-11-15

**Authors:** Hatice Kök, Ayse Merve Acilar, Mehmet Said İzgi

**Affiliations:** 1 Faculty of Dentistry, Department of Orthodontics, Selçuk University [SÜ], Alaeddin Keykubat Campus, Akademi Square, Yeni İstanbul Street 309, Selçuklu, Konya, Türkiye; 2 Engineering and Architecture Faculty, Department of Computer Engineering, Necmettin Erbakan University [NEÜ], Konya, Türkiye; 3 Private Practice, İstanbul, Türkiye

**Keywords:** Artificial intelligence, Algorithms, Cervical vertebrae, Growth and development, Orthodontics

## Abstract

**Background:**

Growth and development can be determined by cervical vertebrae stages that were defined on the cephalometric radiograph. Artificial intelligence has the ability to perform a variety of activities, such as prediction-classification in many areas of life, by using different algorithms, In this study, we aimed to determine cervical vertebrae stages (CVS) for growth and development periods by the frequently used seven artificial intelligence classifiers, and to compare the performance of these algorithms with each other.

**Methods:**

Cephalometric radiographs, that were obtained from 300 individuals aged between 8 and 17 years were included in our study. Nineteen reference points were defined on second, third, and 4th cervical vertebrae, and 20 different linear measurements were taken. Seven algorithms of artificial intelligence that are frequently used in the field of classification were selected and compared. These algorithms are k-nearest neighbors (k-NN), Naive Bayes (NB), decision tree (Tree), artificial neural networks (ANN), support vector machine (SVM), random forest (RF), and logistic regression (Log.Regr.) algorithms.

**Results:**

According to confusion matrices decision tree, CSV1 (97.1%)–CSV2 (90.5%), SVM: CVS3 (73.2%)–CVS4 (58.5%), and kNN: CVS 5 (60.9%)–CVS 6 (78.7%) were the algorithms with the highest accuracy in determining cervical vertebrae stages. The ANN algorithm was observed to have the second-highest accuracy values (93%, 89.7%, 68.8%, 55.6%, and 78%, respectively) in determining all stages except CVS5 (47.4% third highest accuracy value). According to the average rank of the algorithms in predicting the CSV classes, ANN was the most stable algorithm with its 2.17 average rank.

**Conclusion:**

In our experimental study, kNN and Log.Regr. algorithms had the lowest accuracy values. SVM-RF-Tree and NB algorithms had varying accuracy values. ANN could be the preferred method for determining CVS.

## Introduction

One of the basic elements of orthodontic treatment is timing. Skeletal parameters are affected by growth and development, causing changes in the sagittal, transversal, and vertical planes in patients. For individuals with severe orthodontic anomalies, whose growth and development have been completed, orthognathic surgery-assisted orthodontic treatment is recommended [1–3]. Determination of the ideal initiation time of orthodontic and/or dentofacial orthopedic treatment may be as crucial as the selection of the specific treatment regimen [4]. Given the treatment can be initiated in the patient’s optimal growth and development phase with a suitable protocol, the most positive response with the least potential morbidity can be expected [5].

Growth and development can be determined by chronological age, menarche, or changes in voice, anthropometric indicators such as height increase and skeletal maturation (bone age) [6, 7]. Since chronological age alone is not sufficient to fully reflect the actual growth time, various skeletal maturation indicators have been developed [6]. In the determination of growth and development, skeletal maturation stages obtained from radiographic analyses are widely used in order to predict the time of pubertal development, to determine the growth rate, the peak period of growth and the remaining growth and development potential [8, 9]. Traditionally, the gold standard of determining the growth and development periods of individuals was achieved by hand-wrist radiographs. Skeletal maturation (bone age) also can be determined by the help of cervical vertebra maturation stages. In orthodontic practice, the use of cephalometric radiographs for the purposes of diagnosis and establishing treatment plans has gained wide acceptance [10, 11]. Lamparski first reported growth and development could be inferred from the cervical vertebrae. The reliability and validity of the cervical vertebral method combined with the hand-wrist method and have been confirmed by studies conducted in different parts of the world [12, 13]. It is easy to record and interpret the growth and development determination from cephalometric radiographs, and this method also prevents the patient from receiving additional radiation [9].

Due to major advances in technology, computer programs that assist in the diagnosis, treatment, and prognosis are routinely used in the field of orthodontic science. Currently, there are many studies utilizing artificial intelligence and bioinformatics for the purposes of prediction, classification, and clustering of real-life problems [14–17]. Genetic algorithms, expert systems, fuzzy logic, logistic regression, random forest, decision tree, k-nearest neighbors algorithm (k-NN), support vector machine (SVM), Naive Bayes, and artificial neural networks are among the main artificial intelligence algorithms. To our knowledge, no computer-aided method has been developed to determine growth, development, and bone age from cervical vertebrae, a gap in the field that has not been explored. In this study, we aimed to determine cervical vertebrae stages (CVS) for growth and development periods by the frequently used seven artificial intelligence classifiers and to compare the performance of these algorithms with each other.

## Material and methods

Ethical approval for the study was obtained from Necmettin Erbakan University Ethics Committee on Research except for Pharmaceuticals and Medical Device. In our retrospective study, cephalometric radiographs obtained from 300 individuals aged between 8 and 17 years. A total of 10 groups with 30 individuals were included in the study.

Cephalometric radiographs of individuals were taken in the natural head position. All cephalometric radiographs included in this study are radiographs with adequate quality and in which second (C2), third (C3), and fourth (C4) cervical vertebrae are clearly observed. Our subject selection criteria for the study included individuals that were not subjected to trauma and/or operation in the head and neck region, did not undergo orthodontic treatment, did not have any disorder that could interfere with bone development, did not have any systemic disease and/or growth and development retardation, and did not have congenital and/or acquired malformations in the head and neck region.

**Fig. 1:**
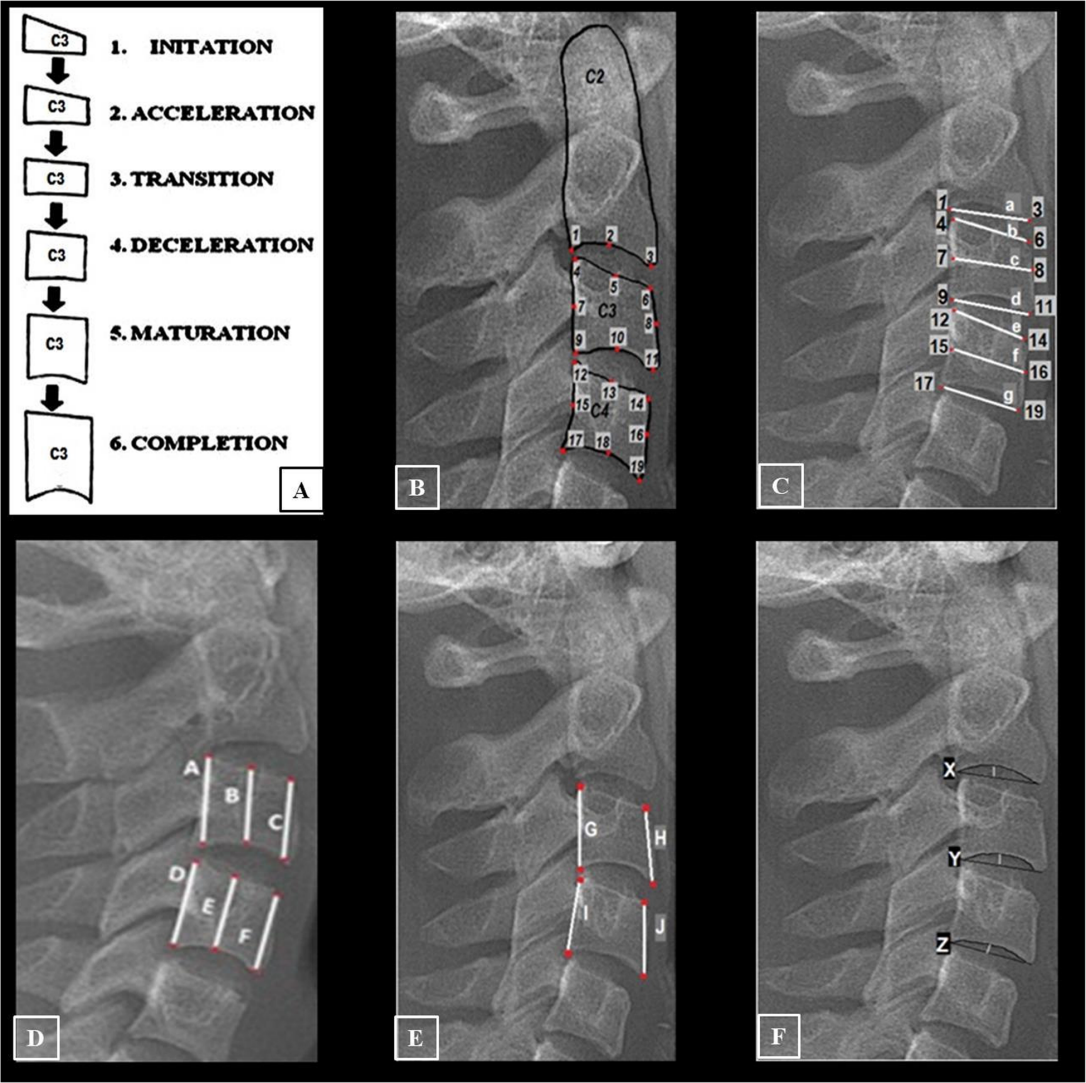
The cervical vertebral references and measurements. **a** The observed alteration of C3.^18^. **b** The cervical vertebral reference points. **c** The horizontal linear measurements. **d** The vertical linear measurements. **e** The anterior and posterior vertebral slope measurements. **f** The vertebral depth measurements

For the determination of growth and development of individuals from cephalometric radiographs, the second (C2), third (C3), and fourth (C4) cervical vertebrae are evaluated and divided into six stages: cervical vertebrae stage 1 (CVS1) is the starting stage during which adolescent growth commences. C2, C3, and C4 are trapezoidal, and their upper edges are inclined forward. CVS2 is the acceleration stage during which adolescent growth is accelerated. Concavity starts at the lower edge of C2 and C3. The lower edge of C4 is flat. C3 and C4 start to look rectangular. CVS3 is the change stage during which there is an adolescent growth spike. At the lower edges of C2 and C3, concavity becomes evident. At the lower edge of C4, concavities start. C3 and C4 assume rectangular morphologies. CVS4 is the deceleration stage during which adolescent growth slows down considerably. At the lower edges of C2, C3, and C4 concavities become evident. C3 and C4 begin to look like a square. CVS5 is the maturity stage during which adolescent growth is not very significant. The concavities at the lower edges of C2, C3, and C4 become more evident. C3 and C4 take the shape of a square. CVS6 is the stage of completion during which adolescent growth is completed. Growth is not expected at this stage. The concavities at the lower edges of C2, C3, and C4 deepen significantly. C3 and C4 are either in a square shape, or their vertical dimensions are larger than the horizontal dimension (Fig. 1a) [18].

**Fig. 2:**
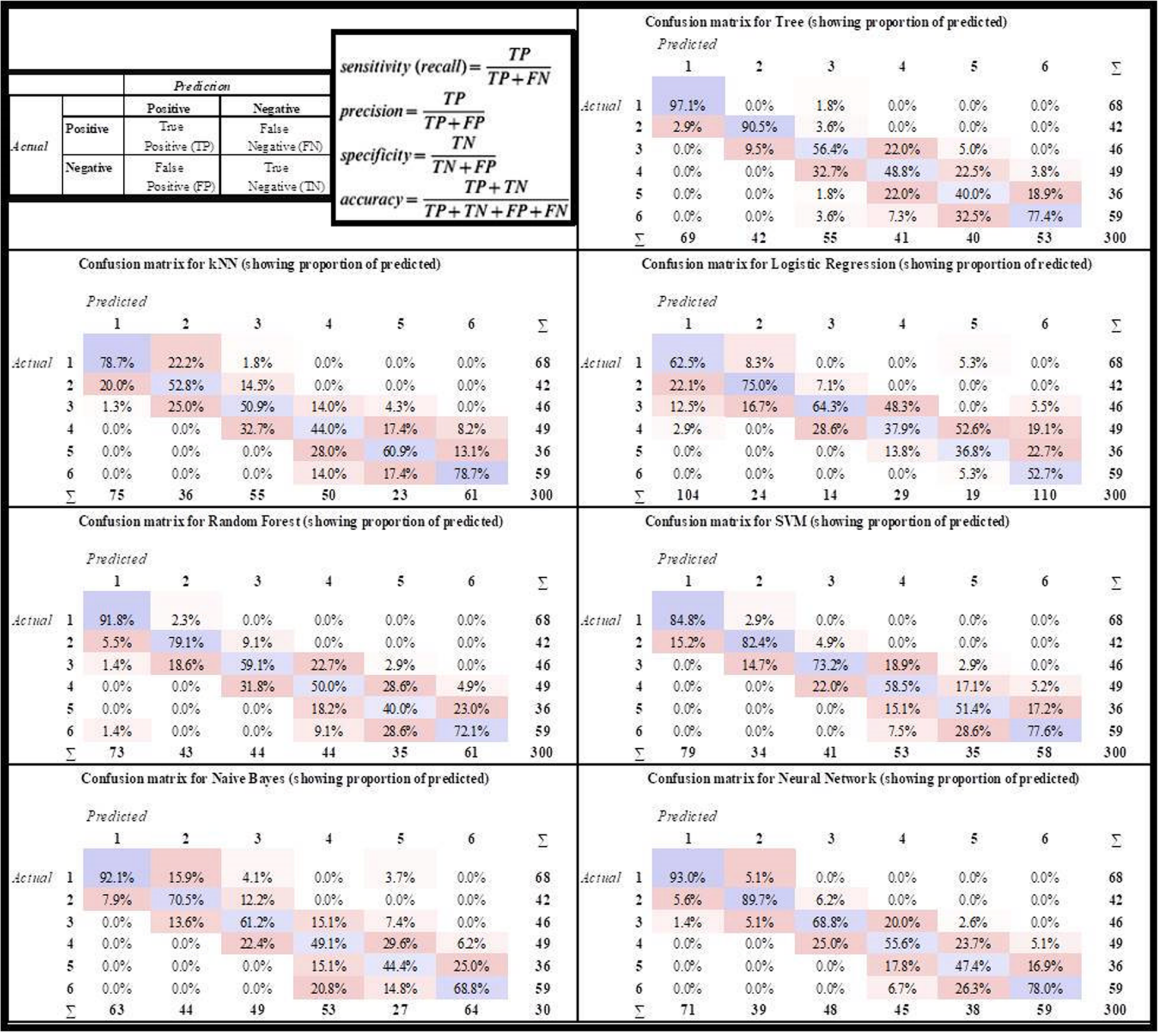
The basic notations and the confusion matrices of the algorithms which were used for determination of CVS

**Fig. 3:**
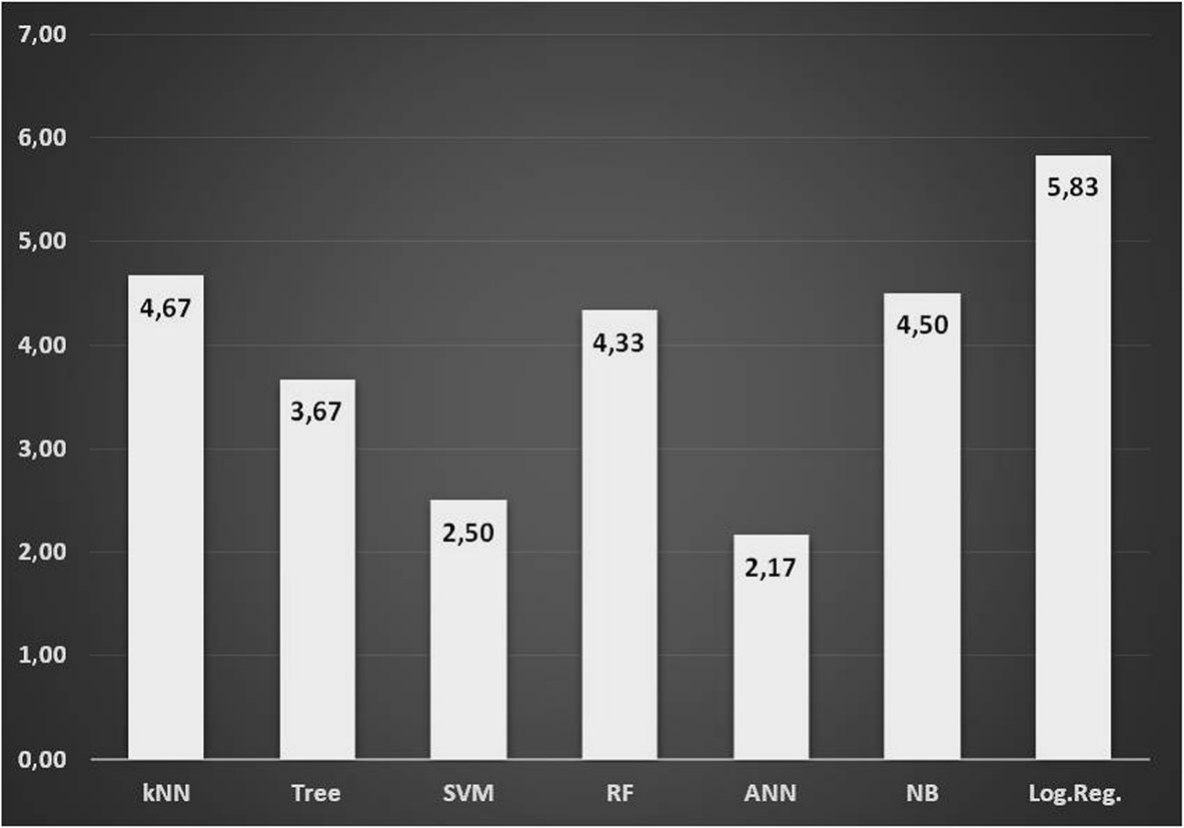
The mean rank values of the algorithms used for determination of CVS according to classification accuracy

**Fig. 4:**
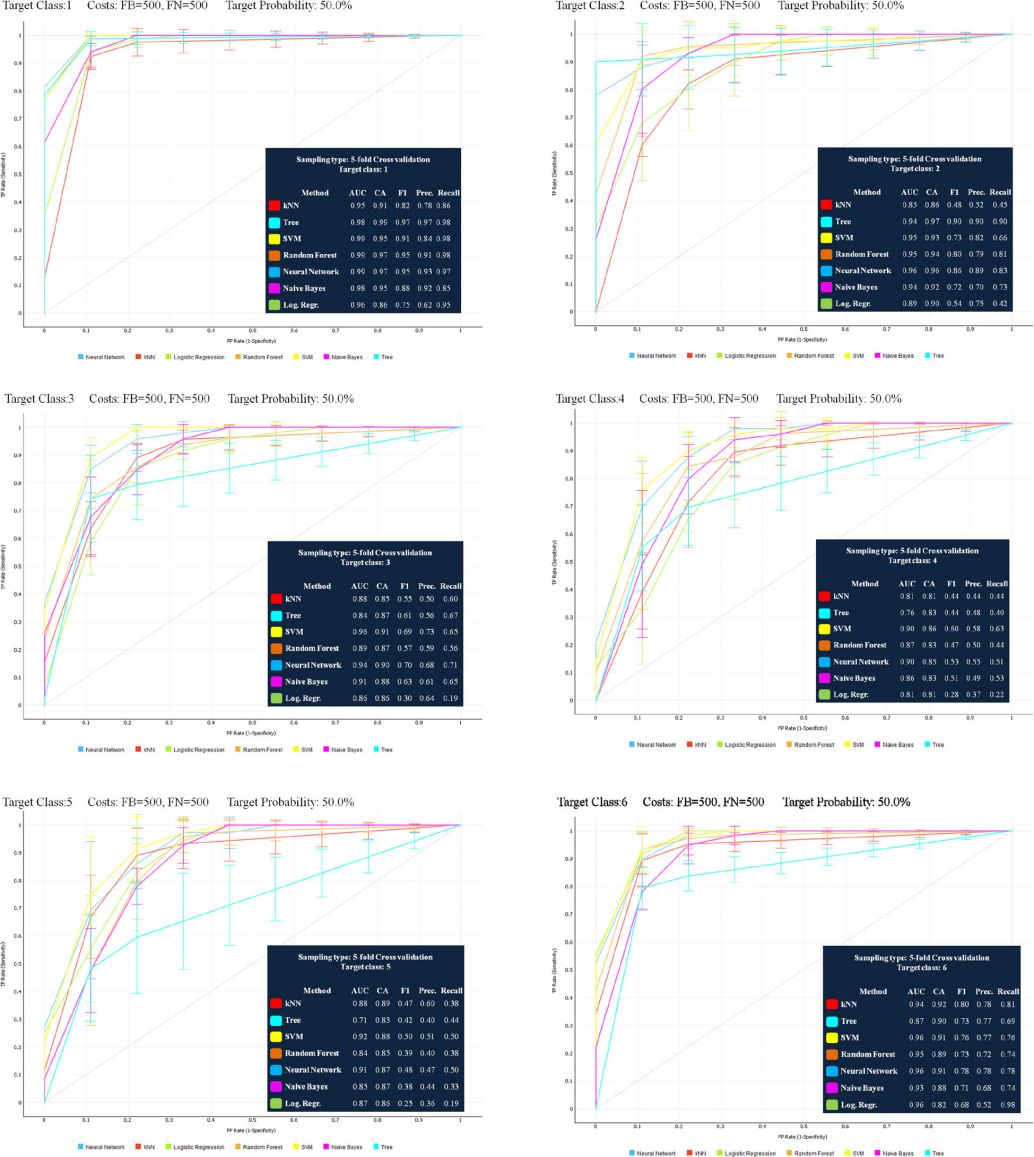
The ROC curves of the algorithms used for determination of CVS and the tables of average test set classification accuracy (CA) results from five-fold cross-validation

The steps of our experimental study were designed as follows: firstly, CVS of each radiograph was determined by the orthodontist. These were labeled as actual CVS. One month later, this process was repeated by the same orthodontist and the intra-examiner reproducibility for the cervical vertebral stages was tested by Fleiss Kappa and a substantial agreement was found (ҝ = 0.7484). Then, 19 reference points were defined on C2, C3, and C4 (Fig. 1b; Table 1). The vertebral measurements were detected according to our literature research. The different researcher’s measurements were combined to comprehensive our study [26, 27, 31, 32]. Thus, a total of 20 different linear measurements were performed on C2–C3 and C4 in each cephalometric radiograph. These were C3–C4 anterior-medial-posterior height, C3–C4 upper-medial-lower width, C2 lower width, C3–C4 slope, and C2–C3–C4 depth measurements (Fig. 1c–f; Tables 2, 3, and 4). The measurement data were given as input to artificial intelligence algorithms, and the predicted CVS as a result of these algorithms were obtained. Then, the predicted CVS was compared with the actual CVS (cross-validation (Fig. 2), rank (Fig. 3), ROC curve (Fig. 4)).

**Table 1:** The cervical vertebrae reference points

No.	Point name	Description
1	SVp	The posterior point of the lower edge of the second vertebra
2	SVd	The deepest point of concavity at the lower edge of the second vertebra
3	SVa	The anterior point of the lower edge of the second vertebra
4	TVup	The posterior point of the upper edge of the third vertebra
5	TVum	The center point of the upper edge of the third vertebra
6	TVua	The anterior point of the upper edge of the third vertebra
7	TVpm	The posterior point of the center edge of the third vertebra
8	TVam	The anterior point of the center edge of the third vertebra
9	TVlp	The posterior point of the lower edge of the third vertebra
10	TVd	The deepest point of concavity at the lower edge of the third vertebra
11	TVla	The anterior point of the lower edge of the third vertebra
12	FVup	The posterior point of the upper edge of the fourth vertebra
13	FVum	The center point of the upper edge of the fourth vertebra
14	FVua	The anterior point of the upper edge of the fourth vertebra
15	FVpm	The center point of the posterior edge of the fourth vertebra
16	FVam	The center point of the anterior edge of the fourth vertebra
17	FVlp	The posterior point of the lower edge of the fourth vertebra
18	FVd	The deepest point of concavity at the lower edge of the fourth vertebra
19	FVla	The anterior point of the lower edge of the fourth vertebra

**Table 2:** The horizontal and vertical linear measurements

The horizontal linear measurements			The vertical linear measurements		
No.	Name	Description	No.	Name	Description
a	SVp-SVa	Point 1 to point 3	A	TVup-Tvlp	Point 4 to point 9
b	TVup-TVua	Point 4 to point 6	B	TVum-Tvd	Point 5 to point 10
c	TVpm-Tvam	Point 7 to point 8	C	TVua-TVla	Point 6 to point 11
d	TVlp-TVla	Point 9 to point 11	D	FVup-Fvlp	Point 12 to point 17
e	FVup-FVua	Point 12 to point 14	E	FVum-FVd	Point 13 to point 18
f	FVpm-Fvam	Point 15 to point 16	F	FVua-FVla	Point 14 to point 19
g	FVlp-Fvla	Point 17 to point 19

**Table 3:** The anterior and posterior vertebral slope measurements

No.	Name	Description
G	TVlp-TVup XY	The slope of the posterior edge of C3 vertebrae relative to the *x* and *y* planes
H	TVua-TVla XY	The slope of the anterior edge of C3 vertebrae relative to the *x* and *y* planes
I	FVlp-FVup XY	The slope of the posterior edge of C4 vertebrae relative to the *x* and *y* planes
J	FVua-FVla XY	The slope of the anterior edge of C4 vertebrae relative to the *x* and *y* planes

**Table 4:** The vertebral depth measurements

No.	Name	Description
X	SVD	The perpendicular distance from “*a*” to deepest point of the inferior border of the second vertebrae
Y	TVD	The perpendicular distance from “*d*” to deepest point of the inferior border of the third vertebrae
Z	FVD	The perpendicular distance from “*g*” to deepest point of the inferior border of the forth vertebrae

The premise behind the use of artificial intelligence is to assist in making more accurate and unbiased decisions, thus increasing the efficiency of systems. Therefore, in the present study, seven algorithms of artificial intelligence that are frequently used in the field of classification were selected and compared. These algorithms are k-nearest neighbors (k-NN), Naive Bayes (NB), decision tree (Tree), artificial neural networks (ANN), support vector machine (SVM), random forest (RF), and logistic regression (Log.Regr.) algorithms.

The k-nearest neighbors (k-NN) algorithm makes classification according to the distance between the instances. The nearest k neighbor to the point to be classified is determined, and according to the status of these neighbors, the class of the new instance is determined. Naive Bayes is a statistical classification algorithm based on the Bayes theorem. Firstly, the frequency and probability tables are created for each instance of *x*. Then, by using these tables, the *P*(*c*|*x*) conditional probability values are calculated for each class of c of a new incoming instance. It is said that instance *x* belongs to the class, for which the highest probability value is obtained. The decision tree is a hierarchical data structure that implements the “divide and manage” strategy. Each attribute is represented by a node. Branches and leaves are the elements of the tree structure. The very last structure is named the leaf, while the top structure is named the root, and the remaining structures between them are named the branch. The entropy values of the attributes and the system will determine which of the attributes will be the root and which of them will be the branch. ID3, ASSISTANT, and C4.5 are examples of decision tree algorithms. Artificial Neural Network (ANN), a mathematical model of the human nervous system, is a system formed by interrelated artificial nerve cells (neurons). The strength of this system is at solving non-linear problems. It can decide on new instances it has never encountered, via establishing connections between the training examples [19]. The support vector machine (SVM) is an artificial intelligence algorithm that separates the attribute space from the hyperspace, thereby aiming to maximize the margin between instances of different classes/class values. The SVM can also be applied to multiple classification problems [20, 21]. Random Forest, an ensembles-learning algorithm, forms a series of decision trees. Each tree is developed from a bootstrap instance from the training data. While trees are formed one by one, a random subset from attributes is generated (the expression of random in the algorithm is originated from here). The class of the new instance is determined by using the majority vote of individual trees in the forest [22, 23]. Logistic regression is a statistical method used to analyze a dataset consisting of one or more independent variables that determine the output. It is the regression model employed when dealing with categorical outcomes (as opposed to continuous outcome where linear regression can be applied). It is the model obtained by adding a regularization term (such as the Lasso regression that was used in this study) to the cost function in order to eliminate the over-learning problem of linear regression [24].

### Statistical analysis

In the present study, a dataset was created by taking vertebral measurements from the cephalometric radiographs of 300 individuals. Descriptive statistics were made for cervical vertebrae stages (CVS) and the distribution of mean ages and age ranges according to their genders (Table 5).

**Table 5:** The descriptive statistics of the males’ and females’ age by CVS

CVS	*N*		Average age (years)		Age range (years	
	Female	Male	Female (*X* ± SD)	Male (*X* ± SD)	Male	Female
1	33	35	8.79 **±** 0.78	9.03 **±** 0.98	8–12	8–10
2	11	31	9.73 **±** 1.01	10.71 **±** 1.13	8–12	8–12
3	18	28	11.17 **±** 0.62	12.86 **±** 0.89	11–14	10–12
4	27	22	12.63 **±** 1.08	14.41 **±** 0.80	13–16	11–15
5	19	17	13.84 **±** 0.76	15.71 **±** 0.47	15–16	13–15
6	42	17	16.05 **±** 0.90	16.88 **±** 0.33	16–17	14–17

In order to determine CVS, which is one of the attributes obtained from cervical vertebral linear measurement values, the relevant algorithms were operated in Orange 3.11 software and the results were evaluated. Five-fold cross-validation was used to evaluate the performance of the algorithms. In the cross-validation process, the dataset is divided into five subsets. One of these subsets is separated as a test set to validate the accuracy of the system, while the remaining four are used as training sets. This process is repeated for each subset. This ensures that each data point is included in the test set at least once. As a result, the average of five operations for the test sets is taken, and the classification accuracy (CA) of the system is calculated. Also, the results can be expressed using the confusion matrix. In the confusion matrix, the lines represent the numbers of the actual class value of the samples, while the columns represent the model’s prediction. Sensitivity (recall), precision, area under the curve (AUC), and F1 criterion were used to measure the model’s success by confusion matrix (Fig. 2).

In statistics, “ranking” refers to the data transformation in which numerical values are replaced by their rank when the data are sorted in descending or ascending order. In our study, we used CA as numerical data and the ranks were assigned to CA values in descending order. For example, CA values are 90.5%, 97.1%, 52.8%, and 78% are obtained, the ranks of these data items would be 2, 1, 4, and 3, respectively.

## Results

In the created study model, the confusion matrices were obtained for the six classes (CVS1-CVS6) by implementing the ANN, k-NN, decision tree, random forest, SVM, and logistic regression algorithms for all data set (Fig. 2).

Decision tree CSV1 (97.1%)–CSV2 (90.5%), SVM CVS3 (73.2%)–CVS4 (58.5%), kNN CVS5 (60.9%)–CVS 6 (78.7%) were the algorithms with the highest accuracy in determining cervical vertebrae stages, while kNN CVS2 (52.8%) and CVS3 (50.9%) had the lowest accuracy. The logistic regression algorithm was observed to have the lowest accuracy with the values of CVS1 (62.5%)–CVS4 (37.9%) and CVS6 (52.7%). The random forest algorithm had the lowest accuracy for CVS5 (36.8%). The ANN algorithm was observed to have the second-highest accuracy values (93%, 89.7%, 68.8%, 55.6%, and 78%, respectively) in determining all stages except CVS5 (47.4%—third highest accuracy value). The average rank of the algorithms in predicting the CSV classes is presented in (Fig. 3). It was found out that ANN was the most stable algorithm with its 2.17 average rank.

In this study, ROC curves were drawn based on average test results obtained from five-fold cross-validation for each vertebral growth and development stage (Fig. 4). As a result of the AUC evaluation, it was observed that the ANN algorithm had the highest values in determining all stages with the exception of CVS3 and CVS5. The SVM algorithm had the highest values in determining the CVS3 and CVS5 stages. As a result of the CA evaluation, the ANN algorithm was determined to have the second-highest value in a stable way of determining all stages.

## Discussion

Uysal et al. [25] evaluated the relationship between chronological age and bone ages determined from hand-wrist and cervical vertebrae radiography with the Spearman rank-order correlation coefficients and reported that cervical vertebrae stages can be used clinically in the determination of growth and development in Turkish individuals. Mito et al. [26] developed a regression formula to determine bone age from cervical vertebrae. Caldas et al. [27] took measurements from the cervical vertebrae and developed a regression formula for Brazilian individuals. Alkhal et al. [28] compared chronological ages, hand-wrist bone ages which were determined according to the TW3 and GP method, and cervical vertebral bone ages which they determined by the formula they developed by using the stepwise multiple regression analysis from cervical vertebra measurements and reported the results to be compatible and highly correlated with each other. Baidas reported that chronological age was a weak indicator of skeletal maturation and that cervical vertebrae could serve as a better indicator for this purpose [29]. In 2010, in order to determine bone age from cephalometric radiographs by using a computerized and semi-automated system, Caldas et al. took measurements by marking the vertebral reference points on the computerized cephalometry program and by also incorporating the formula they derived previously [30]. Alhadlaq and Al-Maflehi [31] presented a statistical method for the determination of bone age from cervical vertebrae by the stepwise multiple regression analysis using chronological age and the ratio between the measurements. Beit et al. [32] evaluated the relationship between morphological changes on cervical vertebrae, the bone age according to the hand-wrist (GP) atlas, and chronological age according to the Bland and Altman plot. On the other hand, Nestman et al. [33] and Gabriel et al. [34] reported that cervical vertebrae staging has very poor reproducibility. Hand-wrist radiographs are accepted as the gold standard at the determination of growth-development, but the definition from the cephalometric radiographs, routine for orthodontic treatment, could be advantageous for both the patients and the clinicians.

Mito et al. [26] Caldas et al. [27, 30], and Alhadlaq and Al-Maflehi [31] performed eight linear measurements on C3 and C4 among cervical vertebrae, while Chen et al. [10] performed eight linear measurements on C2–C3 and C4. Beit et al. [32] performed 10 linear measurements on C2–C3, and C4. These researchers reported that since the first (C1) and fifth (C5) cervical vertebrae could not be observed clearly and given the minimal changes in C2, they were not included in the study. In the present study, although there were minimal changes in C2, we deemed it necessary for C2 data to be included. As a result, 20 linear measurements were taken on C2–C3 and C4.

To our knowledge, although studies have been carried out in order to automatize growth and development determination from cervical vertebrae in orthodontics, no method using artificial intelligence has been encountered. Multidisciplinary studies have been mostly performed on hand-wrist radiographs. Chang et al. [35] described the computer-assisted method of determining bone age based on the characteristics of phalanges, and they benefited from the backpropagation neural network for classification purposes. In this study, 10 input parameters from the left-hand X-ray images were applied to the neural networks, and 77.69% classification success was achieved. Giordano et al. [36] conducted studies on the pineal and diaphyseal images in hand-wrist radiographs in order to determine the bone age automatically. Li et al. [37] introduced the automatic detection of growth and development periods with the TW3 method from hand-wrist radiographs and with ANN among artificial intelligence methods.

According to confusion matrices for all data set, Log.Regr had the lowest values for CVS1–CVS4–CVS5–CVS6. Most of the CVS4 was identified as CVS3 and CVS5 was identified as CVS4 by Log.Regr. algorithm. Its CA values of five-fold cross-validation were low too. It was observed from confusion matrices that kNN had the highest values for CVS5–CVS6 and the lowest values for CVS2–CVS3. By the way, kNN algorithm had low values at five-fold cross-validation. In determining CVS, different algorithms for each stage showed low and high values. In our experimental study, algorithms except ANN were not able to demonstrate consistent achievement in determining each class correctly. The ANN algorithm was observed to have the second-highest accuracy values in both five-fold cross-validation and confusion matrices results for determining all stages except CVS5. We have shown the ANN algorithm has been found out to be the steadier algorithm than others in determining cervical vertebrae stages. According to confusion matrices, ANN classification success was CVS1 (93%), CVS2 (89.7%), CVS6 (78%), CVS3 (68.8%), CVS4 (55.6%), and CVS5 (47.4%), respectively. The five-fold cross-validation of ANN’s CA values were the lowest 85 and higher.

## Conclusions

Artificial intelligence algorithms can be used for diagnostic purposes in all sciences where growth development needs to be determined. Thus, more accurate and unbiased decisions can be obtained. By providing decision support to clinicians, it can provide faster and more effective diagnosis and contribute to the accuracy, reliability, and reproducibility of the diagnosis. In the orthodontic science that is digitized day by day, we think that time and labor can be saved by developing computer-assisted decision support programs and integrating them into existing programs.

As a conclusion, kNN and Log.Regr. algorithms had the lowest accuracy values, while SVM, RF, Tree, and NB algorithms had varying accuracy values so ANN could be the preferred method for determining CVS according to our experimental study.

## Data Availability

The data supporting the findings of this research can be obtained directly from the authors.

## Abbreviations

ANN: Artificial neural networks algorithm
C2: Second cervical vertebrae
C3: Third cervical vertebrae
C4: Fourth cervical vertebrae
CVS: Cervical vertebrae stages
CVS1: Cervical vertebrae stage 1
CVS2: Cervical vertebrae stage 2
CVS3: Cervical vertebrae stage 3
CVS4: Cervical vertebrae stage 4
CVS5: Cervical vertebrae stage 5
CVS6: Cervical vertebrae stage 6
k-NN: k-nearest neighbors algorithm
Log.Regr: Logistic regression algorithm
NB: Naive Bayes algorithm
RF: Random forest algorithm
SVM: Support vector machine algorithm
Tree: Decision tree algorithm
